# Anastrozole plus leuprorelin in early maturing girls with compromised growth: the “GAIL” study

**DOI:** 10.1007/s40618-015-0399-z

**Published:** 2015-10-27

**Authors:** D. T. Papadimitriou, E. Dermitzaki, M. Papagianni, G. Papaioannou, V. Papaevangelou, A. Papadimitriou

**Affiliations:** Department of Pediatric-Endocrinology and Diabetes, Athens Medical Center, 58, av. Kifissias, Maroussi, 15125 Athens, Greece; Third Department of Pediatrics, “Attikon” University Hospital, Haidari, 12462 Athens, Greece; Third Department of Pediatrics, Hippokrateion General Hospital of Thessaloniki, Aristotle University of Thessaloniki, 54642 Thessaloniki, Greece; Department of Radiology, Mitera Maternity and Children’s Hospital, Maroussi, 15123 Athens, Greece

**Keywords:** Aromatase inhibitors, Anastrozole, LHRH analogues, Early puberty, Girls

## Abstract

**Purpose:**

Aromatase inhibitors have been used to increase predicted adult height (PAH) in boys but in girls only in McCune-Albright syndrome. We investigated whether anastrozole combined with leuprorelin for up to 2 years is safe and effective in improving PAH in girls with early puberty and compromised growth, compared to leuprorelin alone.

**Methods:**

The “GAIL” study: girls treated with an aromatase inhibitor and an LHRH analogue, ISRCTN11469487, was a 7-year prospective phase IIa study with parallel design, performed at Athens Medical Center (C-A), and Attikon University Hospital, Athens, Greece (C-B). Forty girls, consecutively referred for early puberty (onset 7.5–9 years) with a PAH <−2 or >1.5 SD lower than their target height (TH), were included. Twenty started on leuprorelin sc/im 0.3 mg/kg/month plus anastrozole 1 mg/d p.o. (group-A, C-A) and 20 on leuprorelin (group-B, C-B) for 2 years or until the age of 10 years. Groups did not differ in age, height, BMI, bone age advancement (BAA), and distance of PAH from TH. Follow-up was at 6, 12, 18, and 24 m.

**Results:**

Reduction in BAA was significantly higher in group-A compared to group-B already by 6 m. Despite the transiently significant decrease in height velocity in group-A, gain in PAH SD was almost double by 12 and 18 m vs group-B and reached the maximum of +1.21 ± 0.45 (7.51 cm) vs +0.31 ± 0.37 (1.92 cm, *p* = 0.001) in group-B at 24 m. Group-A had no clinical or biochemical hyperandrogenism, unchanged normal bone density, and lumbar spine X-rays.

**Conclusion:**

The co-administration of anastrozole with leuprorelin safely improves PAH in girls with compromised growth.

## Introduction

Third generation aromatase inhibitors (AI), mainly anastrozole and letrozole, have been used to increase predicted adult height (PAH) [1, 2] in boys with constitutional delay of growth and puberty (CDGP) [3], idiopathic short stature (ISS) [4–6], and growth hormone deficiency (GHD) [6, 7], as well as in testotoxicosis [8], and gynecomastia [9–11], but in girls only in the context of McCune-Albright syndrome [12–14] apart from one case report of a girl with a recurrent autonomous ovarian cyst [15]. The reluctance of using of aromatase inhibitors in girls stems from the theoretical concern of secondary hyperandrogenism and ovarian cyst formation [16, 17].

To relieve and overcome these concerns, we combined anastrozole with an LHRH analogue in girls with precocious puberty (PP) or early puberty (EP) and compromised growth potential [median PAH 145.4 cm with a median target height (TH) of 161 cm] in a small pilot clinical trial [18]. In that study, 5 girls (aged 6.3–11.5 years) received leuprorelin 11.25 mg/12 weeks s.c./i.m. and anastrozole 1 mg/day p.o. for 1–3 years. There was a net significant increase in PAH of +4.5 cm already by the end of the 1st year. None developed ovarian cysts or other signs of hyperandrogenism or any other systematic side effects, while bone mineral density (BMD) evaluated yearly remained unchanged [DXA scans showed *z* scores (corrected for height) within normal range for BA without significant inter-patient changes].

It has been already shown that in EP and in borderline PP, inhibition of puberty alone is not as effective as initially expected in improving PAH, with most studies showing a gain of 2 cm or less after a 2-year treatment with an LHRH analogue (LHRHa) [19]. On the other hand, atypical forms of early puberty not driven by LH, such as those occurring in cases of premature or exaggerated adrenarche, do not seem to respond to LHRH analogues at least in terms of bone maturation and gain in adult height [20].

These facts led us to plan and implement a phase IIa prospective parallel group study, the “GAIL” study: girls treated with an *A*romatase *I*nhibitor and *L*euprorelin, to investigate whether the combination of anastrozole and leuprorelin could significantly improve PAH in girls with EP and a compromised growth potential, compared to inhibition of puberty alone.

## Materials and methods

The “GAIL” study ISRCTN11469487 was a 7-year prospective study that was performed between 2008 and 2014 in the pediatric endocrine units of the Athens Medical Center (Center-A) and of the Attikon University Hospital (Center-B), Athens, Greece. Inclusion started on March 2008 and ended on December 2013. The institutional review boards of both institutions approved the study. Written informed consent was obtained from at least one parent in group-B and from both parents in group-A, as this was an off-label treatment.

### Inclusion criteria

Forty girls with idiopathic early puberty, i.e. breast development between 7.5 and 9 years [21], with a PAH—calculated according to the Bayley–Pinneau method using the average girls’ prediction tables [22, 23]—lower than −2 or −1.5 SD lower than their TH (mid parental height −6.5 cm), previously naïve to any hormonal therapy, consecutively referred to the above centers, were enrolled for a maximum period of 2 years, or until the age of 10 years [24]. Girls with organic PP, syndromic, systemic or hereditary conditions that either impair growth or associate with PP, as well as bone diseases related to short stature (i.e. hypochondroplasia) ,were excluded from the study. Diagnosis of PP was based upon clinical examination and laboratory evaluation including a positive LHRH test [peak LH ≥5 IU/L at 30′ or 60′ after i.v. administration of gonadorelin 100 μg/m^2^–max 100 μg (Relefact LHRH^®^, Ferring SAS)] associated with elevated basal *E*_2_ ≥ 15 pg/mL and pubertal configuration of the internal genitalia on the pelvic ultrasound: maximum uterine length ≥35 mm, pear shaped (uterine/cervix width >1), ovaries ≥3 mL with the presence of stimulated follicles ≥6 mm in diameter). BA was advanced more than the expected difference in years between their height percentile and their TH percentile, as these girls did not follow the well-recognized pattern of constitutional advancement of growth and puberty (CAG) [25]. Girls in Center-B were offered inhibition of puberty with leuprorelin and those in Center-A inhibition of puberty with leuprorelin combined to the AI anastrozole. Patient characteristics forming group-A and group-B are shown in detail in Table 1.

**Table 1:** Patient characteristics (mean ± SD)

Group	Age (years)	Height (SDS)	BMI (SDS)	TH (SDS)	TH-PAH (SDS)	Bone age advancement (years)
A: LHRHa + AI (*n* = 20)	8.91 (±0.98)	−0.19 (±1.36)	1.15 (±0.89)	−0.27 (±0.80)	−2.17 (±1.00)	1.88 (±1.11)
B: LHRHa (*n* = 20)	8.46 (±0.65)	0.53 (±0.83)	1.13 (±1.08)	0.15 (±0.73)	−1.81 (±0.58)	1.95 (±0.67)
*p*	0.058	0.06	0.47	0.055	0.09	0.40

*LHRHa* LHRH analogue, *AI* aromatase inhibitor, *BA* bone age, *BMI* body mass index, *TH* target height, *PAH* predicted adult height

### Study design

A complete physical examination with accurate height measurements (and of both parents), pubertal Tanner staging, a bone age X-ray, a pelvic ultrasound by a pediatric radiologist, as well as the required biochemical testing (at 0800 h and after an overnight fast) were obtained at inclusion. These studies were repeated on the day of the scheduled injection with leuprorelin every 6 months during follow-up.

Each girl enrolled was consecutively followed by the same experienced Pediatric Endocrinologist: D.T.P in Center-A and A.P. in Center-B. Bone ages were evaluated blindly according to the Atlas of Greulich and Pyle by an experienced Pediatric Radiologist G.P., who did not work in either institution. G.P. did not have access to the previous readings or the growth chart in the patients’ medical files, nor the regimen the patients were receiving apart from their birth dates.

All measurements, BA readings, and PAH calculations were entered, calculated by and analyzed in growth analyser 3.5 (Copyright© 2001–2006, Dutch growth foundation), using the country default growth curves (for height: Papadimitriou A. 1995, for BMI: The Netherlands 1997, for height velocity: British 1996).

At inclusion a blood count, concentrations of lipids, glucose, calcium, phosphate, alkaline phosphatase, liver enzymes, total vitamin D* and parathyroid hormone* as well as LH*, FSH*, testosterone, and estradiol (*ECLIA, Elecsys immunoassay analyzer, Roche) were performed. Sex steroids and 17OH-progesterone levels were measured by liquid chromatography/tandem mass spectrometry (LC/MS–MS). 17OH-progesterone was <1.5 ng/mL in all cases excluding the possibility of late onset congenital adrenal hyperplasia (CAH). Those with a vitamin D deficiency (i.e. total vit D <20 ng/mL) received proper replacement therapy (i.e. 2000 IU cholecalciferol p.o. daily for 2 months and as needed thereafter) [26].

Dual-energy X-ray absorptiometry (DXA) as well as anterior-posterior/lateral X-ray of the lumbar spine was performed at inclusion and yearly thereafter in group-A patients only. BMD was measured at the lumbar spine (L1–L4) by DXA with a Hologic QDR-1000 upgraded unit (Hologic Inc., Bedford, MA, USA) and was expressed as *z* scores (calculated according to the BA and corrected for height). As reference data for BMD were used the ones provided by the manufacturer of the Hologic densitometer, for Caucasians. The lumbar spine X-rays were evaluated by a pediatric radiologist and were re-evaluated each time in comparison with the previous ones in each patient.

Treatment for EP was with leuprorelin acetate 0.3 mg/kg/month with the 3-month 11.25 mg depot s.c./i.m. injection (Elityran^®^) [27]. All patients had an LHRH stimulation test (0′–30′–60′) performed on the day of and prior to the second scheduled leuprorelin injection with measurement of E_2_ levels and evaluation of the internal genitalia maturation in the pelvic ultrasound.

Treatment with anastrozole tablets started simultaneously at a dose of 1 mg once daily p.o. (Arimidex^®^). Patients were followed at 6-month intervals. Parents were advised to report any sign of hyperandrogenism (acne, hirsutism, hair loss) as well as incidents of peculiar behavior. Medication was electronically prescribed and covered by the patient’s social security at 75 %, which assured compliance with the treatment.

### Statistics

Changes in PAH and in the rest of the parameters presented in the results section between baseline and at 6, 12, 18, and 24 months of treatment was compared using only data from the girls that completed each visit. Analysis between groups was performed with the use of the two-sample two-sided *t* test. Significance was set as a *p* value <0.05.

## Results

**Fig. 1:**
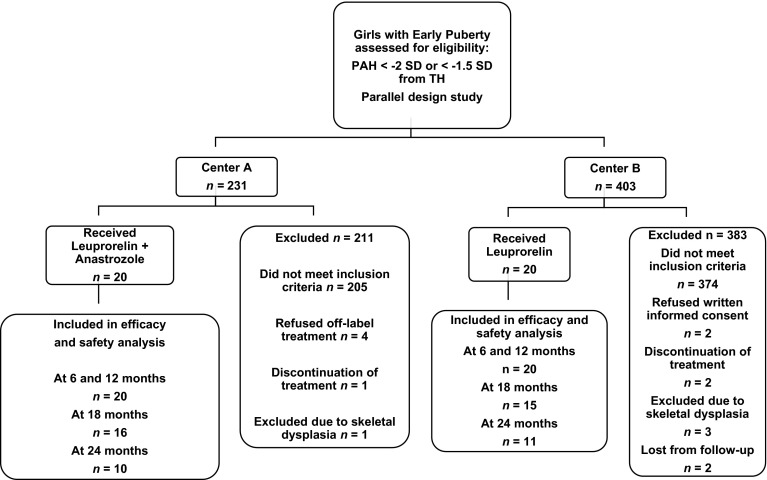
A STARD (STAndards for the Reporting of Diagnostic accuracy studies) flow diagram showing the disposition of subjects participating in the current study

**Fig. 2:**
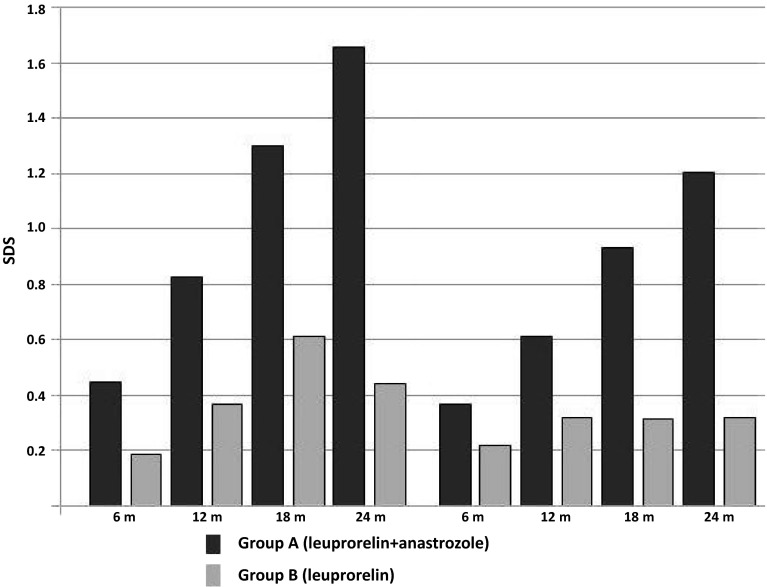
Reduction in bone age advancement (BAA, years: *left side*) and gain in predicted adult height PAH (SDS: *right side*) in group-A (LHRHa + AI) and group-B (LHRHa) at 6, 12, 18, 24 months

The flow chart of the study subjects is shown in Fig. 1. Subjects in group-A (*n* = 20 from Center-A) and group-B (*n* = 20 from Center-B) did not differ in age, height, BMI, BAA, and distance of PAH from TH (Table 1). Evolution of those parameters including also height velocity (HV) is presented in Table 2. Gain in PAH and BAA is presented in Fig. 2. All girls had adequate pubertal inhibition: peak LH <2 IU/L—during an LHRH stimulation test (0′–30′–60′) performed on the day of and prior to the second scheduled leuprorelin injection, *E*_2_ levels <15 pg/mL and regression of breast development as well as of the internal genitalia maturation in the pelvic ultrasound (maximum uterine length <35 mm with the ovaries regressing to <3 mL). The girls on LHRHa only did not present a significant reduction in BA advancement (BAA): +1.82 ± 0.45 at 24 months from +1.95 ± 0.67 years at inclusion (*p* = 0.07). Despite the initial significant drop in HV of group-A at 6 months, which however, did not persist thereafter, there was a significant gain in PAH in group-A at 12 months: +3.72 cm and even more at 18: +5.77 cm and at 24 months: +7.51 cm compared to group-B: +1.98 cm at 12 months (*p* = 0.01), +3.16 cm at 18 months (*p* = 0.02) and +1.92 cm at 24 months (*p* = 0.001) (Table 2). This was achieved because of the significantly higher reduction in BAA of the group-A related to that of the group-B, statistically significant already at 6 months (*p* < 0.001) with a maximum at 24 months (Table 2).

**Table 2:** Evolution (mean ± SD) of height, height for bone age, BMI, height velocity, and bone age advancement in group-A (LHRH analogue + anastrozole) and group-B (LHRH analogue alone)

Group	Height (SDS)		BMI (SDS)		Height velocity (SDS)		Bone age advancement (years)		Reduction in bone age advancement (years)		Target-predicted adult height (SDS)		Gain in predicted adult height (SDS)	
	A	B	A	B	A	B	A	B	A	B	A	B	A	B
Visit 0 *n*(A) = 20 *n*(B) = 20	−0.19 (±1.36)	0.53 (±0.83)	1.15 (±0.89)	1.13 (±1.08)	–	–	1.88 (±1.11)	1.95 (±0.67)	**–**	**–**	2.17 (±1.00)	1.81 (±0.58)	**–**	**–**
***p***	**0.06**		**0.47**		**–**	**–**	**0.40**		**–**	**–**	**0.09**		**–**	**–**
6 months *n*(A) = 20 *n*(B) = 20	−0.29 (±1.32)	0.53 (±0.84)	1.17 (±1.03)	1.05 (±0.93)	−0.19 (±1.24)	0.85 (±1.27)	1.43 (±1.13)	1.77 (±0.63)	**0.44** **(±0.14)**	**0.18** **(±0.42)**	1.8 (±1.00)	1.59 (±0.58)	**0.36** **(±0.17)**	**0.21** **(±0.50)**
*p,* ***p***	0.41	0.49	0.48	0.40	**0.008**		0.12	0.36	**0.009**		0.14	0.42	**0.12**	
12 months *n*(A) = 20 *n*(B) = 20	−0.43 (±1.32)	0.47 (±0.85)	1.01 (±1.50)	0.97 (±0.98)	−1.14 (±1.91)	0.03 (±1.31)	1.05 (±1.11)	1.59 (±0.69)	**0.82** **(±0.28)**	**0.36** **(±0.47)**	1.58 (±0.95)	1.49 (±0.47)	**0.60** **(±0.31)**	**0.32** **(±0.39)**
*p,* ***p***	0.35	0.43	0.36	0.32	**0.051**		0.01	0.059	**<0.001**		0.04	0.039	**0.01**	
18 months *n*(A) = 16 *n*(B) = 15	−0.56 (±1.37)	0.44 (±0.76)	1.11 (±0.83)	1.92 (±0.89)	−1.01 (±2.09)	0.04 (±1.02)	0.42 (±1.09)	1.45 (±0.64)	**1.29** **(±0.32)**	**0.67** **(±0.44)**	1.11 (±0.86)	1.40 (±0.49)	**0.93** **(±0.40)**	**0.51** **(±0.52)**
*p,* ***p***	0.19	0.51	0.37	0.40	**0.043**		<0.001	0.011	**<0.001**		0.004	0.017	**0.02**	
24 months *n*(A) = 10 *n*(B) = 11	−0.97 (±1.36)	0.58 (±0.28)	1.15 (±0.8)	1.47 (±0.77)	−0.99 (±2.96)	−0.03 (±1.46)	0.09 (±1.12)	1.82 (±0.45)	**1.65** **(±0.90)**	**0.44** **(±0.35)**	1,05 (±0.90)	1.45 (±0.36)	**1.21** **(±0.45)**	**0.31** **(±0.37)**
*p,* ***p***	0.08	0.46	0.37	0.49	**0.25**		0.006	0.07	**<0.001**		0.008	0.14	**0.001**	

Reduction in bone age advancement and gain in predicted adult height are reported (*p*: vs visit *0;* ***p***: ***A*** vs ***B***)

Girls in group-A did not develop clinical or biochemical hyperandrogenism or any other side effect. Testosterone levels rose slightly but not significantly and did not exceed 0.5 ng/ml during treatment in any of the girls in group-A: 0.23 ± 0.14 at inclusion, 0.22 ± 0.16 (*p* = 0.92) at 6 months, 0.39 ± 0.34 (*p* = 0.14) at 12 months, 0.32 ± 0.21 (*p* = 0.1) at 18 months, and 0.38 ± 0.35 (*p* = 0.58) at 24 months. None presented ovarian cysts or ovarian stromal hyperplasia and there were no reports of peculiar behavior in any of them. Hematocrit, biochemical, and lipid profiles did not change during treatment. DXA scans showed normal BMD *z* scores for BA: −0.04 ± 1.1 at inclusion, −0.17 ± 1.07 at 12 months (*p* = 0.39), and +0.35 ± 1.14 at 24 months (*p* = 0.24) without significant inter-patient change during treatment. X-rays of the lumbar spine did not show any abnormalities before, during,or at the end of treatment. Basal gonadotropins and estradiol levels were appropriately suppressed (LH <0.2 IU/L, FSH <2 IU/L and *E*_2_ <15 pg/mL) and did not differ between groups at any time point.

## Discussion

With this study we investigated prospectively whether the combination of an AI to an LHRHa could significantly improve PAH in girls with EP and compromised growth, compared to inhibition of puberty alone. Although we acknowledge as a major limitation that this was not a randomized double-blind placebo controlled trial, the study design simulated randomization as much as possible and moreover there was a control group. A parallel study design was chosen, matching each arm of the study with each of the two participating centers as it was judged extremely difficult to obtain parental consent for a potentially off-label treatment with the parents being blind to it. In order to avoid possible biases, every girl who was consecutively referred for pubertal evaluation in each center was assessed for eligibility and only those that met the inclusion criteria and had parental written informed consent entered the study. To increase accuracy, all the parameters presented in the results section were compared between baseline and at 6, 12, 18, and 24 months using only data from those girls that completed each visit and not with the whole of each group at inclusion. We acknowledge the progressive decrease in sample size throughout the study, but at least 50 % of the girls completed 2 years of treatment. This was due to the fact that many reached the age of 10, while at the moment of data analysis still some of the girls had ongoing visits. The results, however, were straightforward showing a net gain of +7.51 cm in PAH at 24 months in the girls treated with the combination therapy vs +1.92 cm with inhibition of puberty alone.

The gain in PAH in the anastrozole group confirmed our preliminary results from the pilot study [18] and was in accordance with the abstract of A.L. Turpin et al. who presented a retrospective study, in which 7 from 19 girls treated with letrozole were concomitantly treated with LHRH analogues [28]. It was also comparable to those of Mauras et al. [7], in adolescent males with GHD and daily co-treatment with anastrozole, that resulted in a net increase in PAH of 6.8 cm at 36 months vs 1 cm in the placebo group. Similar results published recently by Neely EK et al. showed a gain in PAH of 4.2 ± 3.5 cm in peripubertal boys with daily administration of anastrozole vs 1.4 ± 4.4 cm with letrozole after 1 year [5]. While AI have nowadays become an established—even if still an off-label treatment in boys—and this is proven by the fact that recent studies compare directly letrozole to anastrozole in short boys without even a control group [5], to our knowledge this is the first prospective study ever conducted in girls, outside the context of McCune Albright syndrome.

The theoretical concern of hyperandrogenism [16, 17] was overcome with the simultaneous use of LHRHa, keeping the gonadotropins and estradiol adequately suppressed. Thus, the small—even if not statistically significant—rise in testosterone levels could not result in any direct androgenic action on LH regulation [29]. Moreover, recent data on the use of AI as a treatment of infertility in the polycystic ovary syndrome are reasonably reassuring on safety concerns on possible direct deleterious effects on the ovaries [30, 31], whereas measurement of the anti-mullerian hormone levels may indeed be of value but only in the case of monotherapy with AI in girls [32].

The fact that gonadotropins and estradiol levels did not differ between the two groups implies that peripheral aromatization of mainly adrenal but also of ovarian androgens may be the main mechanism of BAA in these girls [1], with extragonadal estrogen biosynthesis, particularly in the bone, deploying a “paracrine” or “intracrine” action [33]. Moreover, the fact that estradiol levels, even if suppressed to below the detection limit in girls treated with leuprorelin for PP, do not correlate with the rate of skeletal maturation or linear growth [34] implies that AI may prove efficacious in delaying bone maturation even in prepubertal children with a compromised growth potential.

There was initially a significantly higher drop in HV in the anastrozole group that did initially raise concerns in some parents but without any implications on compliance. Although this finding did not persist, we speculate that it probably has to do with lower circulating levels of *E*_2_ in group-A, even if still in the prepubertal range. This implies that we may use the least possible dose of an LHRHa if aromatase inhibitors are added. This approach would not only simplify follow-up but may prove cost-effective as well, as intensifying treatment with LHRHa is particularly costly.

Apart from the well-recognized and described pattern of Constitutional Advancement of Growth [35], that obviously does not require treatment, in girls with advanced puberty, borderline PP, precocious slowly progressive and early rapidly progressive puberty, inhibition of puberty alone does not result in significant improvement in terms of height at least after age 8 [19, 36, 37], and moreover its benefits in patients with atypical forms of early puberty not driven by LH (i.e. exaggerated adrenarche followed by early puberty) are not well defined [20]. The co-administration of AI appears to provide the potential for meaningful height gain in girls with borderline precocious or early—rapidly progressive or not—puberty, apart from the individualized possible psychological relief that pubertal inhibition might offer. One might also consider starting an LHRHa alone and add an AI in those cases that BAA does not respond to treatment [18]. It is noteworthy that the maximum gain in the girls treated with leuprorelin only in our study was observed at 18 months (+3.16 cm) and that further continuation of pubertal inhibition may indeed result in loss rather than in further gain as far as PAH is concerned, which is in accordance with Carel et al. [24]. This was not the case in group-A, who continued to ameliorate their PAH at 24 months. In any case the decision on the duration of the intervention must be individualized taking into account the patient’s age, HV, and response of BAA to the combined treatment. Aiming at the lowest normal female adult height or at an SDS lower than the patient’s TH may be an acceptable target with a reasonable rationale from the doctor as well as the patient and their family.

The fact that bone mineral density, hematologic, biochemical, and lipid profiles did not show any changes during treatment suggests that the co-administration of AI with an LHRHa in girls is safe. Moreover, having excluded from the study children with possible bone diseases or abnormalities, the thorough examination of lumbar spine X-rays before and during treatment showed no alterations. This relieves the concern that was raised by Hero et al. [38], who reported the detection of asymptomatic or mildly symptomatic vertebral wedge deformities in boys with ISS previously treated with letrozole vs placebo, although there was no statistical difference between the occurrence rate in the two groups and baseline studies were not performed. In addition, very recently it was reported that more than 20 % of children with ISS present skeletal dysplasia [39].

Further follow-up is needed to examine whether this gain in PAH is translated indeed in higher near-adult height in these girls after discontinuation of treatment.

In conclusion, inhibition of puberty with leuprorelin and co-administration of the third generation aromatase inhibitor anastrozole for up to 2 years is a safe and effective strategy in ameliorating poor PAH in girls with early puberty and a compromised growth potential. Further studies are needed to confirm these results, to evaluate the effect of AI on adult height, as well as long-term safety of the use of AI in girls.

## References

[1] Dunkel L (2006). Use of aromatase inhibitors to increase final height. Mol Cell Endocrinol.

[2] Dunkel L (2009). Update on the role of aromatase inhibitors in growth disorders. Horm Res.

[3] Wickman S, Sipila I, Ankarberg-Lindgren C, Norjavaara E, Dunkel L (2001). A specific aromatase inhibitor and potential increase in adult height in boys with delayed puberty: a randomised controlled trial. Lancet.

[4] Hero M, Norjavaara E, Dunkel L (2005). Inhibition of estrogen biosynthesis with a potent aromatase inhibitor increases predicted adult height in boys with idiopathic short stature: a randomized controlled trial. J Clin Endocrinol Metab.

[5] Neely EK, Kumar RB, Payne SL, Ranadive SA, Suchet DI (2014). Letrozole vs anastrozole for height augmentation in short pubertal males: first year data. J Clin Endocrinol Metab.

[6] Rothenbuhler A, Linglart A, Bougneres P (2015). A randomized pilot trial of growth hormone with anastrozole versus growth hormone alone, starting at the very end of puberty in adolescents with idiopathic short stature. Int J Pediatr Endocrinol.

[7] Mauras N, Welch S, Rini A, Klein KO (2004). An open label 12-month pilot trial on the effects of the aromatase inhibitor anastrozole in growth hormone (GH)-treated GH deficient adolescent boys. J Pediatr Endocrinol Metab JPEM.

[8] Eyssette-Guerreau S, Pinto G, Sultan A, Le Merrer M, Sultan C, Polak M (2008). Effectiveness of anastrozole and cyproterone acetate in two brothers with familial male precocious puberty. J Pediatr Endocrinol Metab JPEM.

[9] Plourde PV, Reiter EO, Jou HC, Desrochers PE, Rubin SD, Bercu BB, Diamond FB, Backeljauw PF (2004). Safety and efficacy of anastrozole for the treatment of pubertal gynecomastia: a randomized, double-blind, placebo-controlled trial. J Clin Endocrinol Metab.

[10] Riepe FG, Baus I, Wiest S, Krone N, Sippell WG, Partsch CJ (2004). Treatment of pubertal gynecomastia with the specific aromatase inhibitor anastrozole. Horm Res.

[11] Shulman DI, Francis GL, Palmert MR, Eugster EA, Drug ftLWPES, Committee T, (2008). Use of aromatase inhibitors in children and adolescents with disorders of growth and adolescent development. Pediatrics.

[12] Mieszczak J, Lowe ES, Plourde P, Eugster EA (2008). The aromatase inhibitor anastrozole is ineffective in the treatment of precocious puberty in girls with McCune–Albright syndrome. J Clin Endocrinol Metab.

[13] Albers N, Jorgens S, Deiss D, Hauffa BP (2002). McCune-Albright syndrome—the German experience. J Pediatr Endocrinol Metab JPEM.

[14] Alves C, Silva SF (2012). Partial benefit of anastrozole in the long-term treatment of precocious puberty in McCune–Albright syndrome. J Pediatr Endocrinol Metab JPEM.

[15] Engiz O, Berberoglu M, Siklar Z, Bilir P, Ocal G (2009). Treatment of autonomous ovarian follicular cyst with long-term anastrozole therapy. Indian J Pediatr.

[16] Mauras N (2009). Strategies for maximizing growth in puberty in children with short stature. Endocrinol Metab Clin North Am.

[17] Geffner ME (2009). Aromatase inhibitors to augment height: continued caution and study required. J Clin Res Pediatr Endocrinol.

[18] Papadimitriou DT, Papadimitriou A (2010) An open label 1–3 year clinical trial on the effects of the aromatase inhibitor Anastrazole combined to the LHRH analogue Leuprorelin in girls with compromised growth potential. Paper presented at the Endocrine Society Meeting, San Diego

[19] Bouvattier C, Coste J, Rodrigue D, Teinturier C, Carel JC, Chaussain JL, Bougneres PF (1999). Lack of effect of GnRH agonists on final height in girls with advanced puberty: a randomized long-term pilot study. J Clin Endocrinol Metab.

[20] Willemsen RH, Elleri D, Williams RM, Ong KK, Dunger DB (2014). Pros and cons of GnRHa treatment for early puberty in girls. Nat Rev Endocrinol.

[21] Papadimitriou A, Pantsiotou S, Douros K, Papadimitriou DT, Nicolaidou P, Fretzayas A (2008). Timing of pubertal onset in girls: evidence for non-Gaussian distribution. J Clin Endocrinol Metab.

[22] Tarim O (2013). Height predictions by Bayley–Pinneau method may misguide pediatric endocrinologists. Turk J Pediatr.

[23] Kirkland JL, Gibbs AR, Kirkland RT, Clayton GW (1981). Height predictions in girls with idiopathic precocious puberty by the Bayley–Pinneau method. Pediatrics.

[24] Carel JC, Chaussain JL (1999). Gonadotropin releasing hormone agonist treatment for central precocious puberty. Horm Res Paediatr.

[25] Papadimitriou A, Kanakis G, Douros K, Papadimitriou DT, Boutsiadis AH, Nicolaidou P, Fretzayas A (2011). Constitutional advancement of growth is associated with early puberty in girls. Horm Res Paediatr.

[26] Hadji P, Aapro MS, Body JJ, Bundred NJ, Brufsky A, Coleman RE, Gnant M, Guise T, Lipton A (2011). Management of aromatase inhibitor-associated bone loss in postmenopausal women with breast cancer: practical guidance for prevention and treatment. Ann Oncol.

[27] Carel J-C, Eugster EA, Rogol A, Ghizzoni L, Palmert MR, Group obotmotE-LGACC (2009). Consensus statement on the use of gonadotropin-releasing hormone analogs in children. Pediatrics.

[28] Turpin ALPJ, Karmazin A, Moore WV, Jacobson JD (2004). Aromatase inhibitor may delay skeletal maturation and improve final adult height in females. Horm Res.

[29] Papadimitriou DT, Linglart A, Morel Y, Chaussain JL (2006). Puberty in subjects with complete androgen insensitivity syndrome. Horm Res.

[30] Legro RS, Brzyski RG, Diamond MP, Coutifaris C, Schlaff WD, Casson P, Christman GM, Huang H, Yan Q, Alvero R, Haisenleder DJ, Barnhart KT, Bates GW, Usadi R, Lucidi S, Baker V, Trussell JC, Krawetz SA, Snyder P, Ohl D, Santoro N, Eisenberg E, Zhang H, Network NRM (2014). Letrozole versus clomiphene for infertility in the polycystic ovary syndrome. N Engl J Med.

[31] Casper RF, Mitwally MF (2012). A historical perspective of aromatase inhibitors for ovulation induction. Fertil Steril.

[32] Winkler N, Bukulmez O, Hardy DB, Carr BR (2010). Gonadotropin releasing hormone antagonists suppress aromatase and anti-Mullerian hormone expression in human granulosa cells. Fertil Steril.

[33] Grumbach MM, Auchus RJ (1999). Estrogen: consequences and implications of human mutations in synthesis and action. J Clin Endocrinol Metab.

[34] Kunz GJ, Sherman TI, Klein KO (2007). Luteinizing hormone (LH) and estradiol suppression and growth in girls with central precocious puberty: is more suppression better? Are pre-injection LH levels useful in monitoring treatment?. J Pediatr Endocrinol Metab JPEM.

[35] Papadimitriou A, Nicolaidou P, Fretzayas A, Chrousos GP (2010). Clinical review: Constitutional advancement of growth, a.k.a. early growth acceleration, predicts early puberty and childhood obesity. J Clin Endocrinol Metab.

[36] Carel JC, Lahlou N, Roger M, Chaussain JL (2004). Precocious puberty and statural growth. Human Reprod Update.

[37] Lazar L, Kauli R, Pertzelan A, Phillip M (2002). Gonadotropin-suppressive therapy in girls with early and fast puberty affects the pace of puberty but not total pubertal growth or final height. J Clin Endocrinol Metab.

[38] Hero M, Makitie O, Kroger H, Nousiainen E, Toiviainen-Salo S, Dunkel L (2009). Impact of aromatase inhibitor therapy on bone turnover, cortical bone growth and vertebral morphology in pre- and peripubertal boys with idiopathic short stature. Horm Res.

[39] Flechtner I, Lambot-Juhan K, Teissier R, Colmenares A, Baujat G, Beltrand J, Ajaltouni Z, Pauwels C, Pinto G, Samara-Boustani D, Simon A, Thalassinos C, Le Merrer M, Cormier-Daire V, Polak M (2014). Unexpected high frequency of skeletal dysplasia in idiopathic short stature and small for gestational age patients. Eur J Endocrinol/Eur Fed Endocr Soc.

